# SARS-CoV-2 Infection Induces a Dual Response in Liver Function Tests: Association with Mortality during Hospitalization

**DOI:** 10.3390/biomedicines8090328

**Published:** 2020-09-04

**Authors:** Vanesa Bernal-Monterde, Diego Casas-Deza, Laura Letona-Giménez, Natalia de la Llama-Celis, Pilar Calmarza, Olivia Sierra-Gabarda, Elena Betoré-Glaria, María Martínez-de Lagos, Lucía Martínez-Barredo, María Espinosa-Pérez, Jose M. Arbones-Mainar

**Affiliations:** 1 Gastroenterology Department, Miguel Servet University Hospital, 50009 Zaragoza, Spain; vbernalm@gmail.com (V.B.-M.); diegocasas8@gmail.com (D.C.-D.); osierra@alumni.unav.es (O.S.-G.); elenab_ejea@hotmail.com (E.B.-G.); 2 Instituto de Investigación Sanitaria (IIS) Aragon, 50009 Zaragoza, Spain; 3 Internal Medicine Department, Miguel Servet University Hospital, 50009 Zaragoza, Spain; laura.letona.g@gmail.com (L.L.-G.); mariamlagos1@gmail.com (M.M.-d.L.); lumbarredo@gmail.com (L.M.-B.); mariespiperez@gmail.com (M.E.-P.); 4 Hospital Pharmacy Department, Miguel Servet University Hospital, 50009 Zaragoza, Spain; ndelallama@gmail.com; 5 Clinical Biochemistry Department, Miguel Servet University Hospital, 50009 Zaragoza, Spain; mpcalmarza@gmail.com; 6 Translational Research Unit, Miguel Servet University Hospital, Instituto Aragonés de Ciencias de la Salud, 50009 Zaragoza, Spain; 7 Centro de Investigación Biomédica en Red Fisiopatología Obesidad y Nutrición (CIBERObn), Instituto Salud Carlos III, 28029 Madrid, Spain

**Keywords:** hepatic biomarkers, COVID-19, mixed models

## Abstract

Severe acute respiratory syndrome coronavirus 2 (SARS-CoV-2) is associated with abnormal liver function tests. We hypothesized that early altered liver biochemistries at admission might have different clinical relevance than subsequent changes during hospitalization. A single-center retrospective study was conducted on 540 consecutive hospitalized patients, PCR-diagnosed with SARS-CoV-2. Liver test abnormalities were defined as the elevation of either gamma-glutamyltransferase (GGT), alanine aminotransferase (ALT), or aspartate aminotransferase (AST), above the upper limit of normality set by our laboratory. Linear mixed models (LMM) evaluated longitudinal associations, incorporating all available follow-up laboratory chemistries. By the end of the follow-up period, 502 patients (94.5%) were discharged (109 (20.5%) died). A total of 319 (64.3%) had at least one abnormal liver test result at admission. More prevalent were elevated AST (40.9%) and GGT (47.3%). Abnormalities were not associated with survival but with respiratory complications at admission. Conversely, LMM models adjusted for age and sex showed that longitudinal increases during hospitalization in ferritin, GGT, and alkaline phosphatase (ALP), as well as a decreased albumin levels, were associated with reduced survival. This dual pattern of liver damage might reconcile previous conflicting reports. GGT and ALP trajectories could be useful to determine who might need more surveillance and intensive care.

## 1. Introduction

Since December 2019, coronavirus disease 2019 (COVID-19) has spread rapidly around the world with high rates of transmission and considerable mortality. As of late August 2020, the number of cases increased to over 23 million, while the death toll surpassed 800,000 worldwide [1]. The emerging severe acute respiratory syndrome coronavirus 2 (SARS-CoV-2) is the causal agent of COVID-19 and evidence proves that liver, among other organs, can be affected by some viruses that primarily target the upper respiratory tract [2,3]. Specifically, liver damage was reported to occur during the infection of another pathogenic coronavirus; the severe acute respiratory syndrome coronavirus (SARS-CoV) and the Middle East respiratory syndrome coronavirus (MERS-CoV) [4,5].

Although primarily a respiratory disease, SARS-CoV-2 infection is also associated with an increased risk for abnormal liver function. Whether this phenomenon is a mere transient biochemistry abnormality, or a surrogate marker of liver injury, is a matter of intense debate. While some authors consider this feature to not to be clinically significant [6,7,8,9,10],⁠ others highlighted its association with adverse outcomes [11,12,13] or reduced survival [14,15,16,17]⁠. This controversy is explained in part due to the lack of a consensus on the definition of COVID-19-associated liver injury [18]⁠. It is, therefore, of paramount importance to generate clinically valid evidence from different populations across the world to account for potential ethnic and geographic variability in COVID-19-related liver injury.

## 2. Experimental Section

### 2.1. Patients and Data Collection

This single center retrospective study was conducted in the Miguel Servet University Hospital, a tertiary care center attending ~400,000 individuals in Zaragoza, in the autonomous community of Aragon (Spain). After obtaining the approval (ref: EPA20/023 on 29 April 2020) from our institutional review board (Comité Ético de Investigación Clínica de Aragón, CEIC-A), we included all consecutive patients that were PCR-diagnosed with SARS-CoV-2 and hospitalized between 5 March 2020 and 8 April 2020. Exclusion criteria were the following—age (<18 years), pregnancy, and the presence of cirrhosis. Clinical data were manually extracted from patients’ medical records. Biochemical tests and drug therapies during hospitalization were directly pulled out from the laboratory and hospital pharmacy data management systems, respectively. Data curation was conducted by the Translational Research Unit of our hospital.

### 2.2. Study Variables and Clinical Complications

Baseline serum laboratory tests were defined as the first test results available upon hospitalization or while the patients were in the emergency room before admission. For variables with missing values, the number of total patients with completed tests is shown. Comorbidities, clinical complications, and outcomes were retrieved from medical records, using ICD-10 definitions.

Liver test abnormalities were defined as the elevation of the liver enzymes in serum, above the upper limit of normality (ULN) set by our laboratory. Those ULNs were as follows—for gamma-glutamyltransferase (GGT), 55 and 38 U/L for men and women, respectively, and for alanine aminotransferase (ALT) and aspartate aminotransferase (AST), these were 50 and 35 U/L for men and women, respectively. Comorbidity burden was measured using the Charlson Comorbidity Index [19]⁠. Acute Respiratory Distress Syndrome (ARDS), chronic kidney disease, sepsis, and multi-organ failure were defined as previously described in [20,21,22]⁠.

### 2.3. Data Analysis

Tests of significance were two-tailed. A stringent alpha level of 0.01 was used to correct for multiple testing. Analyses were performed using the R statistical software (version 3.5.0, https://www.r-project.org/) and appropriate packages.

All data were summarized as median (interquartile range [IQR]), mean (standard deviation), or percentages. Unless otherwise stated, Mann-Whitney and χ^2^ test with Yates correction for continuity were used for pairwise comparison between the continuous and categorical variables, respectively. ANOVA and Kruskal-Wallis tests for comparing 3 groups of parametric and non-parametric variables, respectively, and Wilcoxon signed-rank test to compare each individual’s prior and at-admission biochemistries. The strength of the association among continuous variables was tested by the Pearson’s correlation coefficient. Odds ratios (ORs) and corresponding 95% confidence intervals (CIs) were determined by the univariate logistic regression.

Longitudinal changes for laboratory tests were plotted with the ggplot package, using 1.5 as a smooth parameter (span). Generalized linear mixed models (GLMM) were used to evaluate longitudinal associations incorporating all available follow-up determinations. These models can account for the intra-individual correlations of the repeated measures and can also handle missing data. Intercepts were fitted as random effects to account for inter-individual differences at baseline. Model 1 included the studied laboratory test, time (days since admission), time × laboratory test interaction, age (years), and sex (male or female). The time × laboratory test interaction term indicated differential change between the survivors and deceased in the studied test, from the baseline to the end of the study. The second and third models were additionally adjusted for the treatments with lopinavir/ritonavir (yes or no) and azythromycin (yes or no).

## 3. Results

A total of 540 cases of acute COVID-19 disease were admitted at our hospital during the first peak of virus epidemic in Spain. Of them, a 1-year old child, 2 pregnant women, and 6 individuals with liver cirrhosis were excluded from the analyses. A total of 4.7% of the hospitalized individuals were healthcare personnel. The median age was 70 years (range 22–99) and 47% were female. More women than men were non-smokers (96% vs. 81.2%, respectively, *p* < 0.001) (Table 1). The median interval from symptom onset to hospital admission was 6.5 days. The most common self-reported symptoms at admission were cough (66.5% of patients), dyspnea (61.6%), fatigue (38%), and fever >38 °C or 100.4 F (30.2%). Gastrointestinal symptoms such as diarrhea and anorexia were also cited by 18.8% and 13.7% of patients, respectively. We did not observe differences in the symptom reporting between men and women.

The prevalence of specific comorbidities was 52.2% hypertension, 43.9% dyslipidemia, 22.8% ischemic cardiovascular disease, 22.6% chronic respiratory disease, 16.4% diabetes, 14.9% cancer, 13.9% chronic kidney disease, 11.9% dementia, and 8.8% heart failure (Table 1). The average score on the Charlson Comorbidity Index was 3.8, corresponding to a 59% estimated 10-year survival. Among the laboratory findings, lymphopenia (lymphocyte count < 1.1 × 10^9^/L) and neutrophilia (neutrophil count > 6.5 × 10^9^/L) occurred in 51.3% and 34.8% of patients, respectively. Again, no differences were observed between the sexes in both comorbidities and the white cell counts. Lower median hematocrit was observed in women as compared to men (38.8% vs. 42.2%, *p* < 0.001). SARS-CoV-2 elicited a larger inflammatory response in men, as indicated by the circulating hs-CRP levels (10.5 vs. 7.62 mg/L) for men and women, respectively, *p* < 0.001) and neutrophil to lymphocyte ratio (5.0 vs. 4.0, *p* < 0.001).

### 3.1. Liver Biochemistry at Admission

Laboratory chemistries at admission (presented in Table 2) showed a dramatic decrease of albumin, 58.4% of the performed tests were below the lower threshold of 3.5 g/dL. Men had significantly greater values of ferritin and bilirubin than women.

According to the limits set for GGT, ALT, and AST by the Biochemistry Department in our hospital, 319 patients (64.3%) had at least one abnormal liver test result at admission. No significant differences in the number of abnormal tests were observed between sexes (61.4 vs. 67.7% for men and women, respectively, *p* = 0.478). Test of liver function on admission revealed an elevation of ALT above the normal level in 141 (28.6%) patients (Table 2). More prevalent were the presence of abnormal tests for AST and GGT (40.9 and 47.3%, respectively). Interestingly ALP appeared to be strongly correlated with the GGT values (r = 0.57, *p* < 0.001), suggesting a hepatic origin of the ALP. Neither age nor the Charlson score were significantly associated with the number of abnormalities (Supplementary Figure S1). All the above highlights that the presence of abnormal serum liver function tests was already common at admission, even before the introduction of drug therapies that could worsen the severity of the abnormal serum liver biochemistries.

To rule out a selection bias through which COVID-19 predominately affects patients with elevated liver enzymes, we were able to retrieve laboratory test from 203 patients performed during the 12-month interval previous to admission, which (1) included liver tests and (2) were ordered in primary care settings. As shown in Table 3, COVID-19 infection doubled ALT and AST in men, while a ~50% increase of those two enzymes were observed in women. A 60% post-infection increment of GGT was also observed in both men and women. Neutral effects were observed on serum bilirubin and a ~10% decrease was observed in ALP activity only in women.

### 3.2. Clinical Outcomes of Patients as a Function of Liver Tests at Hospital Admission

As of 15 May 2020, 502 patients (94.5%) were discharged. Median hospital stay was 8 days [interquartile range: 6–12]. During hospitalization, 61 (11.5%) required ICU care and 48 (9.0%) received invasive mechanical ventilation. The number of patients developing each complication was as follows—bacterial pneumonia, 55 (10.4%); acute respiratory distress syndrome, 123 (23.2%); heart failure, 15 (2.8%); arrhythmia, 22 (4.1%); acute myocardial infarction, 2 (0.4%); epileptic seizures, 1 (0.2%); stroke, 9 (1.7%); acute kidney injury, 65 (12.2%); septic shock, 31 (5.8%); disseminated intravascular coagulation, 2 (0.4%); thromboembolic complications, 7 (1.3%); and multi-organ failure, 28 (5.3%). By the end of the follow-up, 109 (20.5%) individuals died.

**Figure 1:**
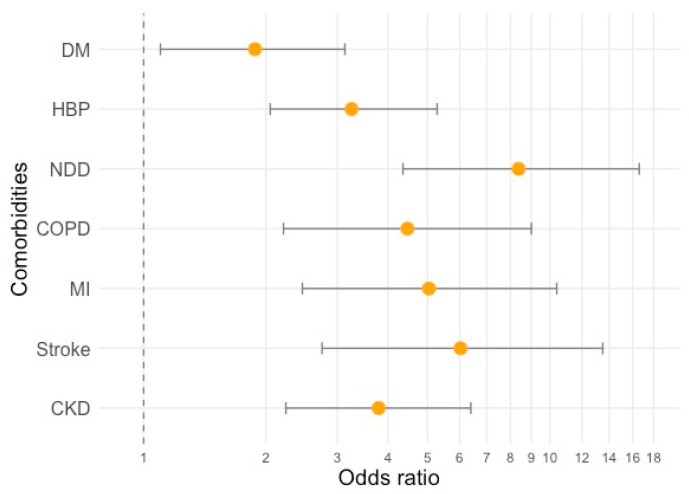
Odds ratios (yellow dots) and 95% confidence intervals for the risk of death during hospitalization, according to existing comorbidities. DM—diabetes mellitus, HBP—high blood pressure, NDD—neurodegenerative diseases, COPD—chronic obstructive pulmonary disease, MI—previous myocardial infarction, and CKD—chronic kidney disease.

Compared to the survivors, non-survivors were significantly older (median age, 65 [53.0; 76.0] vs. 80.0 [74.0; 88.0], *p* < 0.001) and there were more men than women who died (69 men (24.5%) vs. 40 women (16.1%), *p* = 0.022). The youngest deceased patient in our case series was 61-year-old. Death risk was increased by some comorbidities, including diabetes (odds ratio (OR) and [95% confidence intervals] = 1.88 [1.10; 3.13]), high blood pressure (OR = 3.25 [2.05; 5.28]), neurodegenerative diseases (OR = 8.38 [4.35; 16.6]), COPD (OR = 4.46 [2.21; 9.01]), previous myocardial infarction (OR = 5.04 [2.46; 10.4]), stroke (OR = 6.03 [2.75; 13.5]), and chronic kidney disease (OR = 3.79 [2.24; 6.39]) (Figure 1).

Interestingly, the number of abnormal liver tests at admission was not associated with GI but respiratory symptoms. Reduced oxygen saturation, tachypnea, crackles on auscultation, and a trend towards increased dyspnea were more common among individuals with some liver abnormality (Table 4). Moreover, oxygen saturation negatively correlated with ALT (r = −0.18, *p* < 0.001) and AST (r = −0.27, *p* < 0.001) but not with GGT (r = 0.00, *p* = 0.944). This impaired respiratory status translated into more patients with liver abnormalities at admission developing ARDS and requiring ICU care and invasive mechanical ventilation, during their hospitalization. We did not observe, nonetheless, an association between the number of abnormal liver tests at admission and survival.

### 3.3. Longitudinal Effects of Liver Tests on Survival

**Figure 2:**
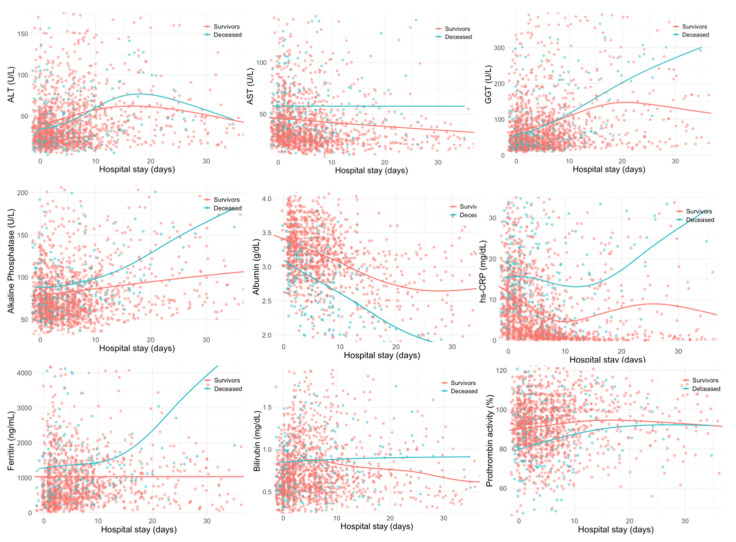
Longitudinal variations for each variable per category (survivors vs. deceased). Scatterplots represent data between the 2.5 and 97.5 percentiles. Each dot represents a single analysis during the first 5 weeks of hospitalization.

During the admission and hospitalization, a total of 1631 laboratory examinations were performed, and the median number of examinations was 3 per patient (range 1–15). Figure 2 depicts all laboratory tests pertaining to liver function and their evolution until discharge or death.

Trends were smoothed using univariate penalized cubic regression splines. Table 5 presents the *p*-values associated with estimates for the change over time for each laboratory test, and whether those longitudinal changes were different, depending on the survival outcomes (interaction). It should be noted that we used a more stringent threshold for statistical significance (*p* < 0.01) to compensate for multiple testing.

**Figure 3:**
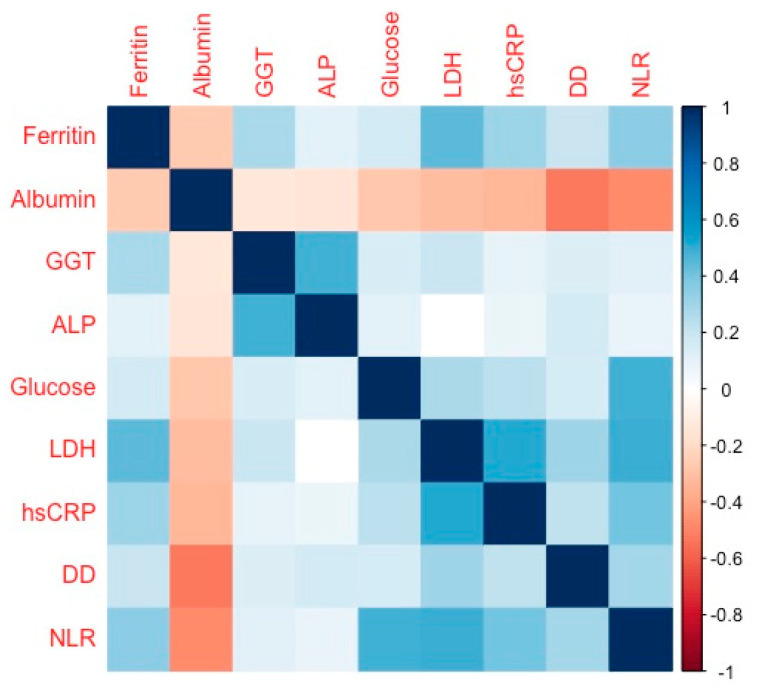
Correlogram illustrating the Spearman correlation coefficients between liver function parameters and inflammatory markers. Color intensity is proportional to the correlation coefficients. White squares denote lack of statistical significance. ALP—alkaline phosphatase; GGT—gamma-glutamyltransferase; LDH—lactate dehydrogenase; hsCRP—high-sensitivity C-reactive protein; DD—D-dimer; and NLR—neutrophil/lymphocyte ratio.

Longitudinal analysis showed that, after adjustment for age and sex, all the above studied parameters except for AST, changed during hospitalization. Interestingly, in the GLMM models adjusted for age and sex the levels of ferritin, albumin, GGT, ALT, and ALP showed a significant interaction between those longitudinal changes and the survival outcomes. This interaction suggests an independent prognostic value for the trajectories of those parameters to predict mortality, even when the models were adjusted for the use of antivirals (lopinavir/ritonavir) or antibiotics (azithromycin), which might cause idiosyncratic liver injury. Thus, an increase during hospitalization in ferritin, GGT, and ALP, as well as decreased albumin levels were the hallmarks for those patients with reduced survival in our cohort. Moreover, the trajectories of ferritin, GGT, and ALP appeared to be positively correlated, while albumin correlated negatively, with glucose and inflammatory markers like hs-CRP, LDH, D-dimer, and neutrophil-to-lymphocyte ratio, during hospitalization (Figure 3).

## 4. Discussion

The COVID-19 pandemic is causing significant increases in mortality across populations [1]. From March to the end of August 2020, Spain has endured more than 46,000 excess deaths compared to previous years [23], resulting in one of the countries with the highest number of deaths relative to its population (~47 million). In this work, we described a dual effect of SARS-CoV-2 infection on liver, characterized by an initial and generalized increase in serum ALT, and AST associated with a reduced oxygen supply. This hypertransaminasemia was in some cases followed by an extensive cholestasis, often associated with poor survival prognosis during hospitalization.

Liver damage is a controversial feature of COVID-19 and its association with clinical outcomes was challenged by some authors [6,10]. For this study, we chose survival during hospitalization rather than other outcomes, such as severity, which is more ambiguous and open to interpretation. We also discarded those patients with preexisting severe liver disease as well children and pregnant women, from the final analyses, as they could confound the liver functionality tests. In addition, we carried out a sensitivity analysis with those patients who had previous liver chemistries tested in primary care setting, to avoid hospitalization bias. This made it possible to (i) rule out that the high levels of transaminases observed at admission were due to the presence of risk factors for liver disease that is frequent in the general population, such as obesity, diabetes, or excessive alcohol consumption, and (ii) it confirmed that hypertransaminasemia is independent of the drug treatment used during patients’ hospitalization. Lastly, we strove to follow the complete clinical trajectories of patients, and this study had one of the highest percentages of discharged patients (~95%) reported to the date.

Liver damage is reported in viral infections that commonly affect the respiratory tract, such as adenovirus, parvovirus, and SARS-associated coronavirus [2,3]. SARS-CoV-2 shares a large genome sequence homology with the other pathogenic human coronaviruses, SARS-CoV and Middle East respiratory syndrome coronavirus (MERS-CoV), both of which are known to cause liver damage [4,5]. At admission, 65% of our patients had an abnormal liver profile defined by an elevation over the ULN for either ALT, AST, or GGT. Specifically, 33% had an elevation of at least 2 liver enzymes, and moderate liver injury (>2 ULN for ALT, AST, or GGT) was observed in 29.2% of the cohort. This prevalence was in line with other US cohorts [16,24] but higher than reported Chinese cohorts [25]. It should be noted that we used the ULN set by our laboratory, which was higher than the “healthy” thresholds recommended by the American Association for the Study of Liver Diseases [26]. Similar to previous studies, we found that AST were more frequently elevated than ALT in patients with COVID-19, with few patients presenting an elevated bilirubin or ALP [27]. On the other hand, serum levels of ALT, AST, and GGT were strongly correlated with hypoxia, in patients with pandemic H1N1 influenza infection [28]. In our study, oxygen saturation was negatively correlated with AST and ALT, but not with GGT. We hypothesized that the observed hypertransaminasemia stems from a reduced oxygen supply associated with the respiratory infection. Alternatively, this elevation might be a mere consequence of an immune response to viral antigens [2]. However, patients with more liver test abnormalities were associated with severe respiratory symptoms (crackles and tachypnea) at admission, and thus primed towards more incidence of ARDS and the need of mechanic ventilation, lending some credit to our hypothesis. A similar transaminase pattern was described previously, in which AST at admission was higher in those requiring ICU care and intubation but failed to predict death [24].

Liver function abnormalities increased during hospitalization; 78.4% of our patients had an abnormal liver profile and transaminase values rising over 2 times the ULN (moderate liver injury) were observed in 38.4% of the cohort. A direct damage was supported by data from an autopsy series of 27 patients that found that SARS-CoV-2 had a tropism beyond the respiratory tract, including the liver, among other organs [29]⁠. This direct effect might occur via intestinal translocation or blood-borne viruses binding to ACE-2 receptors present in the endothelial cells of the liver [30]⁠ or cholangiocytes [31]. GGT is a cholestatic marker that was less frequently reported in the existing COVID-19 case studies, thus far. GGT was reported to be elevated in approximately half of the patients during hospitalization, in two Chinese cohorts [13,32]. In a similar fashion, we found 47% of patients to be above the GGT-ULN, in our cohort at admission. However, this percentage raised up to 60.5% of hospitalized individuals (19.5% over 3 times the ULN). We also found a strong correlation between the longitudinal changes of GGT and the trajectories of ALP (r = 0.64, *p* < 0.001) and bilirubin (r = 0.19, *p* < 0.001), during hospitalization. Although we cannot completely rule out that the increase in ALP was only part of the inflammatory milieu, the joint trajectory of GGT, ALP, and bilirubin points towards a cholestatic liver injury and we found it to be characteristic of individuals with impaired survival. In-patients underwent several drug therapies during hospitalization, mainly hydroxychloroquine/azithromycin, corticosteroids, and lopinavir/ritonavir treatment. However, all our findings remained significant after controlling our statistical models for azithromycin or lopinavir/ritonavir intake, despite the described hepatotoxicity of those regimens [11]. This suggests that drug-induced liver injury did not play a major role in the described pattern of liver injury.

In our cohort, we also found significant changes in the levels of ferritin, albumin, and prothrombin activity, over the hospitalization period. Interestingly, the trajectories of liver function parameters appeared to be highly correlated with inflammatory markers like hs-PCR, LDH, D-dimer, and the neutrophil-to-lymphocyte ratio, during hospitalization. These inflammatory mediators have been repeatedly associated with poor clinical outcomes in COVID-19 [13,33,34]. Our analysis showed that ferritin and hs-CRP, but not prothrombin activity, were differentially upregulated in individuals who died during hospitalization. This was consistent with a scenario where excessive cytokine release caused a dysregulated inflammatory response and multiorgan disease, the so-called “cytokine storm”. On the other hand, hypoalbuminemia was associated with critically ill hospitalized patients [35]⁠ and especially with the most severe manifestations of COVID-19 diseases [36]. In our study, we already noticed lower levels of albumin at admission (median [IQR]: 3.00 [2.80; 3.30] g/dL) in those individuals who died during hospitalization, compared to the survivors (3.40 [3.10; 3.70] g/dL, *p* < 0.001). Albumin levels decreased further for all hospitalized individuals. However, survivors were able to slowly recover admission levels, while albumin plummeted in non-survivors. Although benefits from albumin administration are controversial [37,38], future research should determine whether this treatment might be useful in COVID-19, especially in those elderly patients who tend to have lower albumin levels [39]⁠.

This work has some limitations. First, due its descriptive nature we cannot prove causality. Second, the study mostly included patients with a Southern European ethnicity and its validity in other races/ethnicities needs to be proved. Third, it was a single-center, retrospective study and some cases had missing laboratory chemistries. Lastly, the multiple tests for liver function were carried out at different time intervals for each patient, and patients with more severe diseases might need an increased number of tests.

## 5. Conclusions

The conclusions for this work are manifold. The described dual pattern of liver damage could reconcile previous conflicting reports. ALT and AST elevation at admission might be a consequence of a respiratory impairment and not be associated with poor prognosis, while up-regulated GGT and ALP trajectories during hospitalization were associated with liver injury and decreased survival. GGT and ALP could, therefore, be useful biomarkers for stratifying a population by risk, to determine who might need more surveillance and intensive care. Larger studies are warranted to validate these results and define the role of liver tests in diagnostic algorithms. Lastly, whether this hepatic damage would have repercussions after hospital discharge should also be addressed by follow-up studies.

## Figures and Tables

**Table 1:** Demographic characteristics, comorbidities, and laboratory tests at hospital admission.

Variables	Median [IQR] or Number (%)	*n*
**Demographic**		
Female	249 (46.9%)	531
Age (y)	70.0 [56.5; 79.0]	531
Southern European Ethnicity	484 (91%)	531
Hospital Staff	25 (4.71%)	531
Non-smokers	468 (88.1%)	531
**Comorbidities**		
High blood pressure	277 (52.2%)	531
Obesity (BMI > 30 kg/m^2^)	43 (8.11%)	529
Dyslipidemia	233 (43.9%)	531
Diabetes	87 (16.4%)	531
Dementia	63 (11.9%)	531
Ischemic cardiovascular disease:	121 (22.8%)	531
Chronic respiratory disease:	120 (22.6%)	531
Hearth failure	47 (8.85%)	531
Chronic kidney disease	74 (13.9%)	531
Cancer	79 (14.9%)	531
**Laboratory tests**		
Neutrophil count (×10^9^/L)	4.90 [3.58; 6.70]	488
Lymphocyte count (×10^9^/L)	1.00 [0.70; 1.40]	488
Neutrophil to lymphocyte ratio	4.50 [2.77; 8.30]	488
Hematocrit (%)	40.2 [36.8; 43.3]	488
Glomerular filtration rate (GFR)	82.4 [58.5; 95.4]	492
Lactate dehydrogenase (LDH, U/L)	307 [234; 412]	444
High-sensitivity C-reactive protein (hs-CRP, mg/L)	9.13 [3.79; 15.8]	493

Ischemic cardiovascular disease includes acute myocardial infarction, angina pectoris, ischemic stroke, and peripheral arterial disease. Chronic respiratory disease includes COPD, bronchitis, asthma, and apnea–hypopnea syndrome.

**Table 2:** Baseline liver tests.

		*n*
Albumin (g/dL)	3.38 (0.43)	221
Ferritin (ng/mL)	622 [310; 1219]	269
Alkaline phosphatase (U/L)	73.0 [58.0; 94.0]	493
Bilirubin:		462
Normal	401 (86.8%)	
1–2 ULN	58 (12.6%)	
2–3 ULN	3 (0.65%)	
ALT:		493
Normal	352 (71.4%)	
1–2 ULN	103 (20.9%)	
2–3 ULN	27 (5.48%)	
>3 ULN	11 (2.23%)	
AST:		445
Normal	263 (59.1%)	
1–2 ULN	139 (31.2%)	
2–3 ULN	30 (6.74%)	
>3 ULN	13 (2.92%)	
GGT:		493
Normal	260 (52.7%)	
1–2 ULN	116 (23.5%)	
2–3 ULN	50 (10.1%)	
>3 ULN	67 (13.6%)	

Data are median [IQR], mean (SD), or number of cases (%). ALT—alanine aminotransferase; AST—aspartate transaminase; GGT—gamma-glutamyltransferase; ULN—upper limit of normal; and *n*—number of available cases for each variable.

**Table 3:** Liver tests before and after COVID-19 infection by sex.

	Men (*n* = 100)			Women (*n* = 103)		
	At Admission	Previous Tests	*p*	At Admission	Previous Tests	*p*
Bilirubin (mg/dL)	0.74 [0.56; 0.94]	0.66 [0.54; 0.83]	0.108	0.58 [0.46; 0.76]	0.54 [0.43; 0.65]	0.100
ALP (U/L)	70.5 [56.8; 90.0]	76.5 [62.2; 95.8]	0.109	75.0 [60.0; 96.0]	84.0 [73.0; 109]	0.017
AST (U/L)	46.0 [30.0; 68.0]	23.5 [19.0; 28.2]	<0.001	33.5 [26.0; 46.0]	22.0 [17.0; 25.0]	<0.001
ALT (U/L)	41.0 [24.0; 56.2]	21.5 [14.0; 29.0]	<0.001	25.0 [16.0; 34.0]	18.0 [13.0; 24.0]	<0.001
GGT (U/L)	48.5 [30.8; 110]	29.5 [21.0; 48.2]	<0.001	42.0 [23.0; 85.0]	26.0 [16.0; 40.0]	<0.001

Data are median [IQR]. ALP—Alkaline phosphatase; ALT—alanine aminotransferase; AST—aspartate transaminase; GGT—gamma-glutamyltransferase. *p*: *p*-value for the comparison of groups using the Wilcoxon signed-rank test, to compare each individual’s prior and at-admission biochemistries.

**Table 4:** Symptoms at admission and clinical outcomes by the number of liver abnormalities. Data represent number of cases (%) for the categorical variables and mean (SD) or mean (IQR) for the continuous parametric and non-parametric variables, respectively. *p*: *p*-value for the comparison of groups; chi^2^ for categorical variables and ANOVA or Kruskal-Wallis for continuous parametric and non-parametric variables, respectively; and *n*—number of available cases for each variable.

	Number of Liver Abnormalities				
	0	1	2–3	*p*	*n*
**Symptoms at admission**					
Ageusia	15 (8.67%)	5 (3.38%)	11 (6.63%)	0.151	487
Anosmia	10 (5.78%)	4 (2.70%)	9 (5.42%)	0.377	487
Diarrhea	30 (17.1%)	27 (17.9%)	37 (22.2%)	0.452	493
Crackles	83 (48.3%)	78 (51.7%)	105 (64.4%)	0.008	486
Dyspnea	95 (54.3%)	99 (65.1%)	108 (64.7%)	0.069	494
Tachypnea	24 (13.8%)	45 (30.2%)	40 (24.1%)	0.002	489
Oxygen saturation (%)	95.0 [93.0; 97.0]	94.0 [90.0; 96.0]	93.0 [90.0; 95.0]	<0.001	492
**Clinical outcomes**					
Onset of symptoms to admission (days)	6.51 (10.8)	6.13 (7.09)	7.35 (4.62)	0.385	489
Hospital Stay (days)	7.50 [5.00; 12.0]	8.00 [5.50; 11.0]	9.00 [6.00; 13.2]	0.087	471
ICU care	10 (5.65%)	12 (7.89%)	32 (19.2%)	<0.001	496
Invasive mechanical ventilation	7 (3.95%)	7 (4.64%)	27 (16.2%)	<0.001	495
Bacterial Pneumonia	14 (7.91%)	14 (9.27%)	20 (12.0%)	0.434	495
Acute respiratory distress syndrome	25 (14.1%)	32 (21.2%)	49 (29.3%)	0.003	495
Hearth failure	4 (2.26%)	2 (1.32%)	4 (2.40%)	0.787	495
Arrhythmia	3 (1.69%)	4 (2.65%)	8 (4.79%)	0.256	495
Acute myocardial infarction	1 (0.56%)	1 (0.66%)	0 (0.00%)	0.758	495
Epileptic seizures	1 (0.56%)	0 (0.00%)	0 (0.00%)	1.000	495
Stroke	2 (1.13%)	3 (1.99%)	2 (1.20%)	0.800	495
Acute kidney injury	16 (9.04%)	15 (9.93%)	18 (10.8%)	0.864	495
Septic Shock	4 (2.26%)	6 (3.97%)	14 (8.38%)	0.025	495
Disseminated intravascular coagulation	0 (0.00%)	1 (0.66%)	1 (0.60%)	0.540	495
Thromboembolic complications	4 (2.26%)	2 (1.32%)	1 (0.60%)	0.823	495
Multi organ failure	5 (2.82%)	5 (3.31%)	10 (5.99%)	0.284	495
Death	27 (15.3%)	33 (21.7%)	29 (17.4%)	0.305	496

**Table 5:** *p*-values associated with generalized linear mixed models (GLMM) analysis.

		Interaction between Longitudinal Variation and Survival Groups		
	Longitudinal Variation during Hospitalization	Model 1	Model 2	Model 3
Ferritin	**<0.001**	**<0.001**	**<0.001**	**<0.001**
Albumin	**<0.001**	**<0.001**	**<0.001**	**<0.001**
Bilirubin	**0.008**	0.032	0.073	0.048
GGT	**<0.001**	**<0.001**	**<0.001**	**<0.001**
AST	**<0.001**	0.463	0.463	0.639
ALT	0.013	**0.008**	**<0.001**	**0.009**
Alkaline Phosphate	**<0.001**	**<0.001**	**<0.001**	**<0.001**
hs-CRP	**<0.001**	0.043	0.045	0.037
Prothrombin Activity	**<0.001**	0.101	0.097	0.112

ALT—alanine aminotransferase; AST—aspartate transaminase; GGT—gamma-glutamyltransferase; and hs-CRP—high-sensitivity C-reactive protein. Model 1: Adjusted for age and sex. Model 2: Adjusted for age, sex, and lopinavir/ritonavir treatment. Model 3: Adjusted for age, sex, and azithromycin treatment. Bold characters indicate significant *p*-values (*p* < 0.01).

## Supplementary Materials

The following are available online at https://www.mdpi.com/2227-9059/8/9/328/s1. Figure S1: Number of liver abnormalities according to age (A) and Charlson score (B).
