# Supplementary figures and images for: SARS-CoV-2 Infection Induces a Dual Response in Liver Function Tests: Association with Mortality during Hospitalization
Source: Biomedicines. 2020 Sep 4;8(9):328. doi: 10.3390/biomedicines8090328 (PMC7555293; doi:10.3390/biomedicines8090328)

Supplemental Figure 1. Number of liver abnormalities according to age (A) and Charlson score (B)

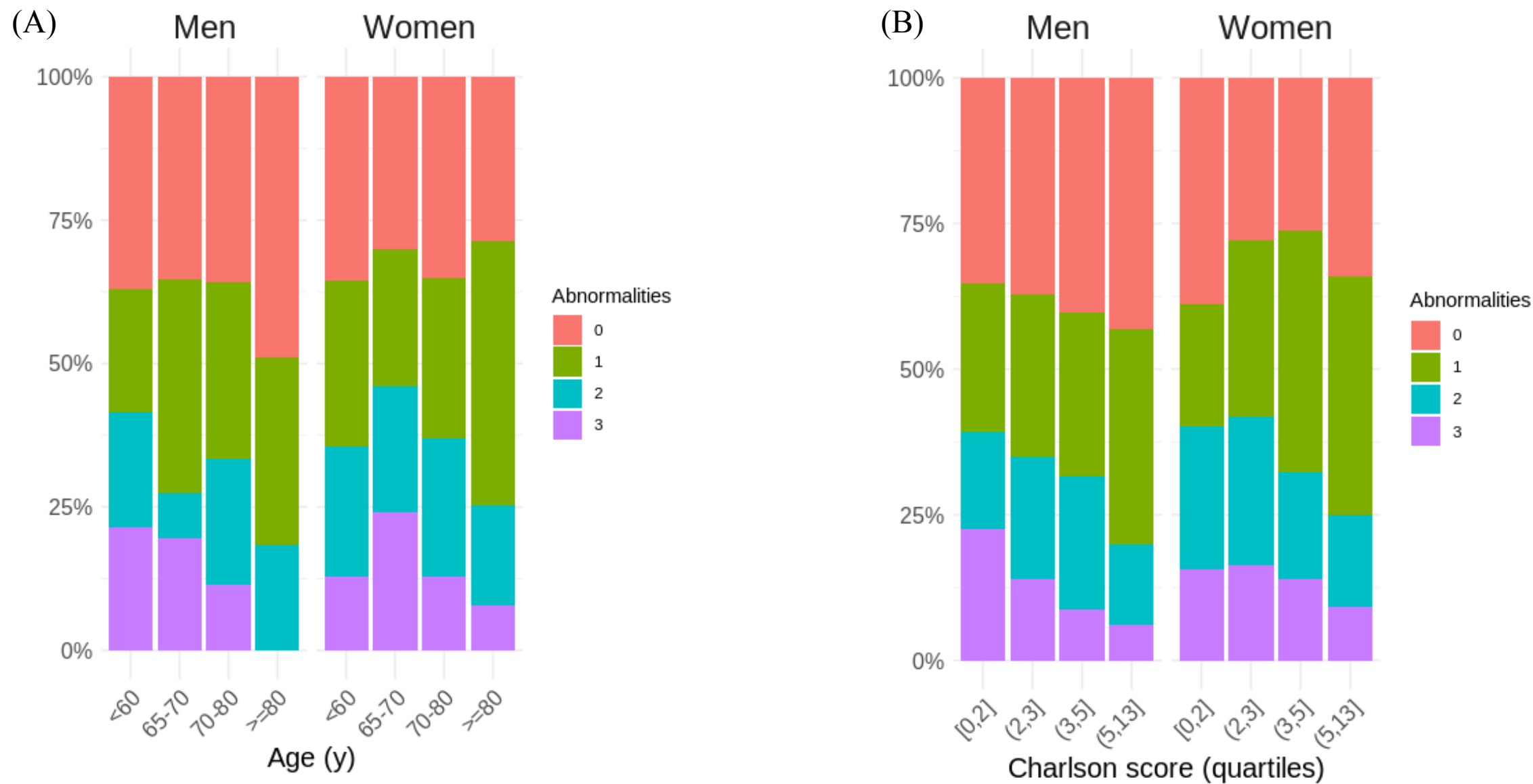

Supplement: Supplementary file 1 [file biomedicines-08-00328-s001.pdf]
